# A Lightweight Network for Point Cloud Analysis via the Fusion of Local Features and Distribution Characteristics

**DOI:** 10.3390/s22134742

**Published:** 2022-06-23

**Authors:** Qiang Zheng, Jian Sun, Wei Chen

**Affiliations:** 1 State Key Laboratory for Strength and Vibration of Mechanical Structures, School of Aerospace Engineering, Xi’an Jiaotong University, Xi’an 710049, China; yongzhoucaomin@stu.xjtu.edu.cn (Q.Z.); cw0523@stu.xjtu.edu.cn (W.C.); 2 Shaanxi Engineering Laboratory for Vibration Control of Aerospace Structures, Xi’an Jiaotong University, Xi’an 710049, China

**Keywords:** lightweight network, deep learning, point cloud classification, point cloud segmentation

## Abstract

Effectively integrating the local features and their spatial distribution information for more effective point cloud analysis is a subject that has been explored for a long time. Inspired by convolutional neural networks (CNNs), this paper studies the relationship between local features and their spatial characteristics and proposes a concise architecture to effectively integrate them instead of designing more sophisticated feature extraction modules. Different positions in the feature map of the 2D image correspond to different weights in the convolution kernel, making the obtained features that are sensitive to local distribution characteristics. Thus, the spatial distribution of the input features of the point cloud within the receptive field is critical for capturing abstract regional aggregated features. We design a lightweight structure to extract local features by explicitly supplementing the distribution information of the input features to obtain distinctive features for point cloud analysis. Compared with the baseline, our model shows improvements in accuracy and convergence speed, and these advantages facilitate the introduction of the snapshot ensemble. Aiming at the shortcomings of the commonly used cosine annealing learning schedule, we design a new annealing schedule that can be flexibly adjusted for the snapshot ensemble technology, which significantly improves the performance by a large margin. Extensive experiments on typical benchmarks verify that, although it adopts the basic shared multi-layer perceptrons (MLPs) as feature extractors, the proposed model with a lightweight structure achieves on-par performance with previous state-of-the-art (SOTA) methods (e.g., MoldeNet40 classification, 0.98 million parameters and 93.5% accuracy; S3DIS segmentation, 1.4 million parameters and 68.7% mIoU).

## 1. Introduction

With the wide application of various three-dimensional (3D) sensors, as a basic 3D data format, point clouds are frequently appearing in many actual scenes, including 3D modeling [1,2,3,4,5,6,7], indoor navigation [8], and autonomous driving [9,10]. Therefore, the demand to understand the shapes represented by 3D point clouds through deep neural networks has gradually emerged.

Unlike the regular grid structure of the two-dimensional (2D) image, the point cloud is a set of unordered spatial points, making it unsuitable for the convolution operation based on the local patch arranged by the grid index. Although deep learning networks such as CNNs have obtained remarkable achievements in processing 2D images [11,12,13,14], it is non-trivial to directly transplant the success of CNN to 3D point cloud analysis. Some alternative approaches have been proposed to alleviate this critical issue. Some methods such as [15,16,17,18,19,20,21] attempt to transform the point cloud into regular voxel format in order to apply 3D CNNs and inherit the advantages of 2D CNNs. However, the performance of voxelization methods is primarily limited by the cubical growth in the computational cost with the increase in resolution. There are also other methods such as [22,23,24,25,26] to render the point cloud from multiple perspectives to obtain a set of images to directly introduce the CNN to process 3D point cloud analysis, which leads to spatial information loss and causes difficulties for tasks such as semantic segmentation.

Since the conversion of point clouds causes information loss and brings extra burdens on storage and computation, it is feasible to directly use the point cloud as the input of deep networks. The point cloud analysis network closely follows the development of image processing technology. The core problem of point cloud analysis is aggregating and extracting features within the perceptual field. There are many methods devoted to designing sophisticated local feature extraction modules, representative works are MLP-based (such as PointNet [27] and PointNet++ [28]), convolution-based (PointConv [29]), graph-based (DGCNN [30]), relation-based (RPNet [31]), and transformer-based (point transformer [32]; PCT [33]) methods. These methods have contributed to the advancement of the point cloud analysis community. However, the pursuit of sophisticated local feature extractors also has its limitations. Delicate modules often correspond to complex modules, resulting in huge computational costs, which hinders the application of these methods to 3D point cloud analysis. In addition, the performance gains from more sophisticated extractors have also been saturated recently. Experiments in [34] show that under similar network frameworks, the performance improvements brought by most refined local feature extractors are not significantly different. Therefore, this paper aims to design a lightweight and efficient network instead of pursuing more refined feature extractors.

**Figure 1:**
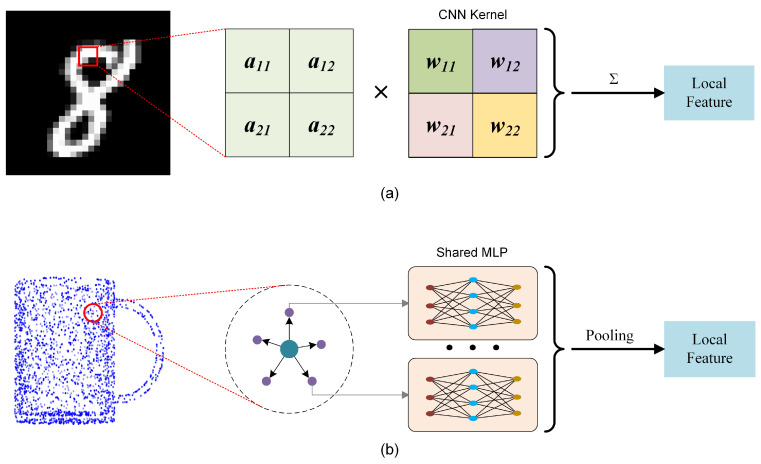
Two local feature extraction methods: (**a**) The convolution kernel provides different weights for input features at different locations, potentially encoding the spatial distribution information of the input features; (**b**) Points at different locations share the same MLP feature extractor, so shared MLP ignores the spatial distribution information.

It is feasible to rethink MLP-based methods, analyze their inherent limitations, and modify them to improve feature extraction capabilities significantly. The advantage of simple MLP-based methods is that they do not require complex operations such as building graphs to extract edge features or generating adaptive convolution kernels. In addition, shared MLP regards all points in the receptive field as equivalent, extracts point features, and then obtains local aggregated features through a symmetric function, which makes the MLP-based methods less computationally expensive and can well adapt to the disorder of point clouds. However, treating all points as equivalent tends to ignore the difference in the spatial distribution, which leads to the deterioration of features. Looking back at the process of CNN using convolution kernels to perform convolution operations on the image patch to extract local features, the weight values in the convolution kernels are usually different, which means that pixels at different positions in the feature map correspond to different weights. Even if the features at different locations are the same, different activation values will be output due to different weights, so the distribution characteristics of elements in the local area also have a potential impact on the extraction of local features. Local features are not only the aggregation of input features in the local receptive field but also potentially encode the spatial distribution information of each element in the local area, see Figure 1a. When performing MLP-based point cloud analysis, shared MLP is usually implemented with a 1 × 1 convolution kernel, which is equivalent to forcing the weights of the convolution kernels corresponding to each position in the local area of the 2D image to be the same. Thus, the feature extraction is independent of the relative position of the pixels, which seriously weakens the feature extraction ability of the convolution kernel, see Figure 1b.

Typical MLP-based methods such as PointNet [27] first extract features for each point independently and then aggregate over the global receptive field to obtain shape-level features. In addition to ignoring local geometric properties, PointNet [27] completely ignores spatial distribution information when aggregating all point features. PointNet++ [28] is an upgraded version of PointNet [27], PointNet++ [28] uses mini-PointNet to aggregate point features in local areas, and only splices the features and relative coordinates in the input layer of mini-PointNet, so PointNet++ [28] does not inherently overcome the limitations of PointNet [27]. Although DGCNN [30] is a graph-based method, it still uses the MLP operation for local feature extraction. DGCNN [30] is aware of the role of spatial distribution information, so it adds the relative coordinates of the neighborhood points and the absolute coordinates of the centroid to the input, which makes the local features depend on the absolute position of the centroid, thus reducing the representation ability of the local features. In addition, DGCNN [30] dynamically builds graphs in Euclidean space and feature space, which introduces much computational consumption. Although CNN-based methods can extract local characteristics based on the spatial distribution information of points, the process of adaptively learning convolution kernels is still computationally expensive compared to MLP-based methods. A concise and effective way to alleviate the limitation of shared MLPs ignoring spatial distribution information is to explicitly provide the relative coordinates of the current receptive field at each MLP layer (for PointNet [27], the receptive field is the entire point cloud, so global coordinates need to be provided). Although this modification is simple, experiments validate its effectiveness. This design allows us to achieve outstanding performance with fewer layers and a more lightweight network.

**Figure 2:**
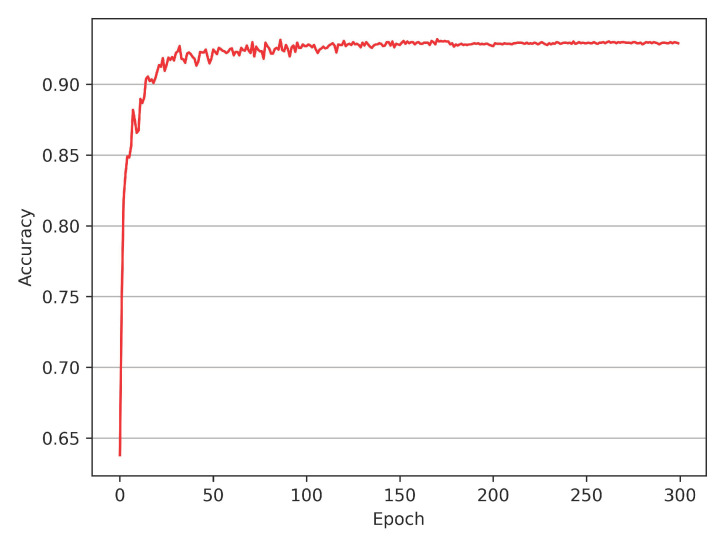
Accuracy curve (red) of the proposed model on the ModelNet40 classification task with an exponential decay learning schedule. Our model converges rapidly and steadily.

Moreover, the vanilla version of the proposed model with an exponential decay learning schedule exhibits rapid convergence, see Figure 2, which implies that performance gains are limited for most epochs beyond the initial growth, accompanied by unnecessary cost of computing resources. We introduce the snapshot ensemble technology to the proposed model to address this issue. Snapshot ensemble can integrate a series of trained models in one complete training session without additional computation cost and fully utilize the model’s rapid convergence to improve the performance further. However, for the snapshot ensemble, the commonly used cosine annealing learning rate cannot be flexibly adjusted when the annealing cycle is fixed, so we propose a novel learning schedule denoted as Rectified-Tanh (Re-Tanh) with an adjustable parameter that can flexibly adapt to different scenarios. Ablation studies also demonstrate that the learning strategy is beneficial to improving the performance of the ensemble model.

The main contributions of this paper can be summarized below:

- We take PointNet [27] and PointNet++ [28] as examples to study the difference in mechanism between shared MLP and CNN convolution kernels, analyze the defects of shared MLP, and reveal that the distribution information in the perceptual field is critical for feature extraction.
- We propose a lightweight structure based on supplementing distribution information to extract discriminative features in a concise manner.
- We introduce the snapshot ensemble to the model and propose a flexible learning schedule to improve the performance.
- We evaluate our model on typical tasks, and the model with the minor parameters achieves on-par performance with the previous SOTA methods. Particularly, the models for ModelNet40 classification (93.5% accuracy) and S3DIS semantic segmentation (68.7% mIoU) have only about 1 million and 1.4 million parameters, respectively.

## 2. Related Work

### 2.1. Methods Based on 3D Voxel

Due to the success of CNN in the field of image processing, it is an intuitive attempt to represent the point cloud with a regular voxel grid. VoxNet [16,17] uses two states of 0 and 1 to indicate whether the voxel is occupied. In order to reduce over-fitting, the approach in [23] predicts the corresponding partial subvolumes of the voxelized data from various directions and uses orientation pooling to fuse the shape features with different directions and converts the analysis of 3D voxels into the extraction of 2D features. The approach in [35] designs a voxel-based variational autoencoder, and the obtained features are used for shape recognition. Voxel-based methods have achieved excellent performance in object recognition tasks, but it has inherent shortcomings. The 3D point cloud is distributed on a 2D manifold, so the 3D voxel data obtained by voxelizing the 2D manifold is highly sparse, and some fine-grained information is lost due to quantization. Moreover, the size of voxel data grows cubically with the resolution, which restricts a higher resolution of voxel data. All these factors will cause a considerable consumption of storage and computation resources. Some methods [19,20,21,36,37,38] have been used to alleviate these difficulties, but they still cannot fundamentally eliminate these limitations. The recently proposed Minkowski Engine [39] is an extension of sparse convolutional networks to high-dimensional space, which facilitates the use of deep networks commonly used in 2D vision for point cloud analysis. Minkowski Engine [39] significantly reduces the storage and computing requirements, enabling voxel-based methods to be applied to higher resolution voxel inputs. Following paper [39], some works [40,41,42] based on Minkowski Engine have demonstrated excellent performance in point cloud analysis.

### 2.2. Methods Based on Multi-View

Another strategy for transplanting the success of CNN to the point cloud is to render the point cloud into images from multiple perspectives, thereby transforming the point cloud analysis into 2D image processing. The method in [22] uses multiple paralleled shared CNNs to process each view and then uses the view-pooling mechanism to fuse features from multiple views to obtain global features. The approach in [23] extracts partial subvolume features of voxel data from spherical projections from multiple perspectives and then designs an orientation pooling layer for generating global features. Ref. [43] aggregates similar features from multi-views through a recurrent clustering and pooling module, which enhances the recognition performance of multi-view 3D objects. In [44], a framework with multi-view attention-convolution pooling utilizes Res2Net to extract the features from multiple views to alleviate the information loss and strengthen the connection between these views. MVTN [45] designs a multi-view transformation network to exploit the optimal viewpoint s adaptively. FSDCNet [46] proposes a view selection method based on fixed and random views to alleviate overfitting caused by the typically fixed viewpoints. Although the efficiency is improved relative to 3D CNN, the conversion of a complete object into a set of discrete rendering images by projection brings about the loss of spatial distribution information and additional requirements for storage resources, and the view-based methods are non-trivial to be extended to the point cloud segmentation tasks, which restricts the further development of such methods.

### 2.3. Methods Based on Point

Unlike converting point clouds into other formats, some methods have emerged in recent years that directly use point clouds as input. PointNet [27] is a pioneering work in such methods. PointNet [27] extracts the features of each point through shared MLPs and aggregates the features of each point through a symmetric function to obtain shape-level features. Since the point feature is directly transferred to the shape feature, the geometric characteristics of the local regions are ignored, which limits the performance of PointNet [27]. Starting from PointNet [27], new methods have been proposed to pursue more delicate feature extraction methods in order to explore more fine-grained geometric features. PointNet++ [28] is an enhanced version of PointNet [27]. PointNet++ [28] focuses on local feature extraction and aggregates local features through hierarchical networks and enlarged receptive fields. PointWeb [47] uses the difference between the point pairs to weigh the corresponding edges in local regions, thereby extracting more fine-grained features. PCNN [48] designs a learnable parameterized kernel function that can adaptively operate on non-grid-structured data. A-CNN [49] proposes multiple local regions composed of concentric rings and design rules to determine the order of points inside the regions to run CNN-like convolution operations to extract local features. AdaptConv [50] generates adaptive kernels based on dynamically learned features to improve the graph convolution in local regions. DGANet [51] builds local dilated graph-like regions and dilated graph attention modules to extract local features for point clouds. OECNN [52] proposes an orientation-encoding convolution module by searching for the same points in eight directions and arranging them to exploit the local features. GAPointNet [53] embeds a graph attention mechanism in cascaded MLP layers to exploit local geometric characteristics in point clouds.

In addition to features within local regions, long-range dependencies between local regions are also important. Besides the typical methods that indirectly obtain inter-region relationships through hierarchical networks and enlarged receptive fields, some methods simultaneously learn intra-region and inter-region relationships. The shape-oriented CNN [54] simultaneously learns the intra-shape relationship inside each local region and also learns the inter-shape relationship by capturing the long-range dependence between the potential local shapes, which enhances the effectiveness of the features. Point2SpatialCapsule [55] designs two modules named geometric feature aggregation and spatial relationship aggregation to explicitly capture the geometric characteristics of local regions and the spatial relationship between them. Point2Node [56] dynamically integrates the correlation between nodes and itself, local and non-local nodes in a high-dimensional graph, and designs a data-aware gate mechanism to aggregate the learned features adaptively. SK-Net [57] proposes an end-to-end framework that jointly optimizes the learnable key points and the extraction of features and then integrates local features and their spatial distribution information to perform point cloud analysis. GS-Net [58] groups distant points with similar and relevant geometric characteristics to aggregate information from nearest feature vectors in both Euclidean space and Eigenvalue space, which can integrate point features from a global perspective. Hybrid-CNN [59] proposes a novel convolution operation named HyConv to capture richer local features and obtain hybrid distribution information in both spatial and feature domains. FFPointNet [60] designs a module named ChannelNet to exploit global shape features and fuses the local and global features for better contextual representation. CGFM-Net [61] proposes a local geometric feature modulation (GFM) block to learn local contextual features and designs a novel global fusion mechanism to preserve the structural information.

Our experiments show that shared MLP is sufficient to process point clouds with high performance compared to refined feature extractors while retaining the advantage of an MLP with low computational complexity and low space complexity. In addition, inspired by CNN and those point-based methods that pay attention to the spatial distribution relationship between regions, our network explicitly provides the spatial distribution information of features by supplementing the coordinates in the receptive field, which enables us to obtain performance on par with the SOTA methods with a lightweight network.

## 3. Methodology

In this section, we first introduce the methods of feature extraction and the supplement of distribution information, then review the snapshot ensemble and illustrate the proposed annealing schedule, and finally, we illustrate the network architecture in detail.

### 3.1. Extracting Local Features

#### 3.1.1. K-Nearest Neighbor Points Search

For a 2D image, a local region of a particular pixel contains pixels located within a certain Manhattan distance from the central pixel that can be determined directly by the index. For a point cloud, a collection of unordered points without a regular grid structure, it is not feasible to directly determine the neighboring points with the index. The general method is *k* nearest neighbor (kNN) search which outputs a fixed number of nearest points around the centroid point in metric space.

Generally, a point cloud containing *N* points can be regarded as a point set denoted as $P=\{p_{1},p_{2},\ldots ,p_{N}\}$, in which an element $p_{i}=\left(x_{i},f_{i}\right)$ contains a point with its 3D coordinates $x_{i}\in R^{3}$ and additional point feature vector $f_{i}$ such as normal, RGB and so on. For kNN research, the input point set is a N×(d+C) tensor where *N*, *d*, and *C* represent the number of points, the dimensions of coordinates and features, and the outcome is a point set of size $N^{{}^{\prime }}\times k\times \left(d+C\right)$, where $N^{{}^{\prime }}$ is the number of sampling points and *k* is the number of neighboring points in each local region.

#### 3.1.2. Local Feature Extraction

**Figure 3:**
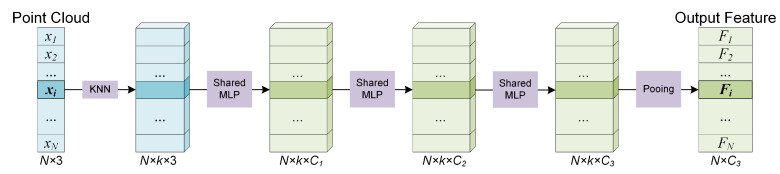
The module for extracting local features based on MLP.

When the kNN search is completed, the neighboring points of a certain centroid point $x_{i}$ are denoted as $N(x_{i})$. Our experiments found that shared MLP, even simple, is sufficient to obtain outstanding performance. Therefore, we choose shared MLP used in PointNet [27] and PointNet++ [28] as the local feature extractor in the trade-off between performance and model complexity. Given a neighbor set $N(x_{i})$ with *k* neighbor points $\left\{x_{i1},x_{i2},\ldots ,x_{ik}\right\}$, the coordinates of points are firstly translated into local coordinates relative to the centroid point to make the local patterns independent of its spatial location. The local feature extractor for centroid *i* can be defined as:

(1) $$F\left(x_{i}\right)=MAX\left\{h\left(x_{ij}-x_{i}\right)\right\},j\in \left[1,k\right],$$

where MAX and *h* represent the max-pooling operation and the stacked shared MLPs, respectively. See Figure 3.

### 3.2. Supplement of the Distribution Information

From the analysis in Section 1, it can be seen that for MLP-based methods, one of the main limitations is to regard points as equivalent and ignore the influence of point distribution on features. Therefore, it is vital to provide information about the spatial distribution of points within the receptive field to improve network performance. We adopt a concise manner by directly providing spatial coordinates as supplementary information to the intermediate layers of the network. The network automatically learns local features and spatial distribution characteristics simultaneously and fuses them to obtain aggregate features. Compared with the previous methods, the proposed model does not require an additional process of establishing complex data structures such as graphs and trees and avoids adding extra modules containing massive trainable parameters to extract distribution information, making our network effective and computationally efficient.

**Figure 4:**
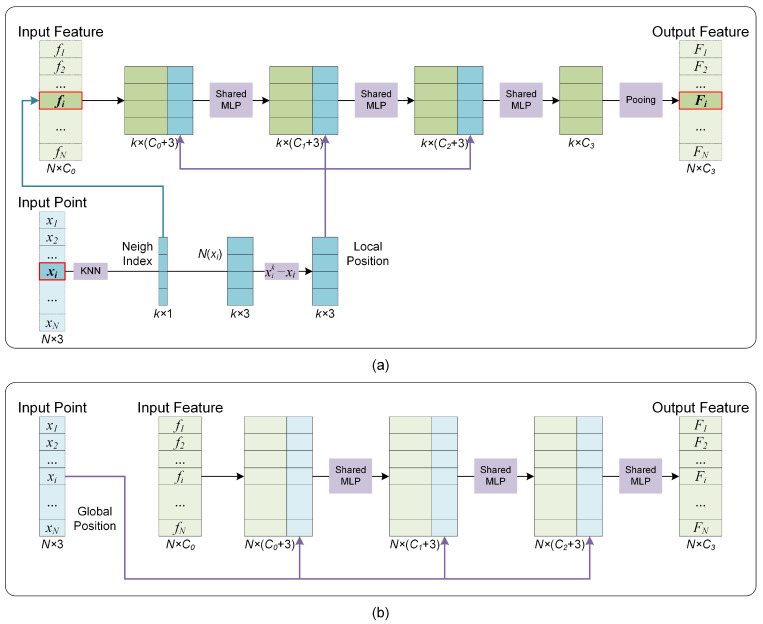
The Fusion Modules corresponding to local (**a**) and global (**b**) perception fields.

Since the supplement mechanism of distribution characteristics involves the fusion of features and their spatial distribution, the corresponding network module is denoted as the Fusion Module. There exist local and global receptive fields in point clouds, so the supplement mechanisms of distributional characteristics are also different. Figure 4 shows the Fusion Modules utilized in this paper. Figure 4a,b correspond to the Fusion Modules with the local and global perception fields. The main difference between the two modules is that the Fusion Module with local perception fields needs to explore the neighborhood points through kNN operation and then calculate the relative coordinates, and the Fusion Module with the global perception field only needs to provide the global coordinates directly. Figure 4b can be seen as a particular case of Figure 4a when the local receptive field is expanded to the entire point cloud.

### 3.3. The Proposed Learning Schedule for Snapshot Ensemble

Model ensemble technology is much more robust and accurate than individual networks. The ordinary method is to train several individual models and fuse the outputs of each model, which is computationally expensive. Snapshot ensemble [62] is an effective method that can ensemble multiple neural networks without additional training cost. Snapshot ensemble follows a cyclic annealing schedule to converge to multiple local minima. The training process thus is split into *M* cycles, and in each cycle, the learning rate starts with a higher value and gradually decreases to a lower learning rate. Starting with a higher value gives the network sufficient momentum to escape from a local minimum, and the subsequent smaller learning rate guarantees the network to converge smoothly to a new local minimum. The general form of the learning rate is:

(2) $$Lr\left(x\right)=\left(lr_{max}-lr_{min}\right)\times f\left(mod\left(t,T\right)\right)+lr_{min},$$

where $lr_{max}$ and $lr_{min}$ are the initial and final learning rate in one cycle, *t* is the iteration number, *T* is the total number of training iterations in one cycle, and *f* is a monotonically decreasing function. In general, *f* is set to be the shifted cosine function:

(3) Shifted-cosine(t)=0.5·cos(πt/T)+0.5.

The experiments reveal that the non-ensemble model rapidly converges when trained with an exponential decay learning rate, see Figure 2. Rapid convergence implies that the proposed model can quickly reach a local minimum in several epochs, facilitating the introduction of the snapshot ensemble.

However, when *T*, $lr_{max}$ and $lr_{min}$ are fixed, the commonly used cosine annealing learning schedule is also fixed, which makes it unable to be flexibly adjusted to adapt to diverse scenarios. Thus we need to design a function that decreases monotonically from 1 to 0 on the interval [0,π] like the shifted cosine, and the shape of the function can be flexibly adjusted. As shown in Equation (4), the tanh function increases monotonically, and we introduce a new annealing schedule based on it.

(4) $$tanh\left(x\right)=\frac{e^{x}-e^{-x}}{e^{x}+e^{-x}}.$$

The steps to rectify tanh to generate a new annealing curve are displayed as follows:

- Use −x instead of *x* to obtain a monotonically decreasing function $f_{1}$:
  (5) $$f_{1}\left(x\right)=tanh\left(-x\right).$$
- The *x* is replaced by sx to scale $f_{1}$, and then $f_{1}$ is truncated on the interval [−π/2,π/2] to obtain the function $f_{2}$:
  (6) $$f_{2}\left(x\right)=tanh\left(-sx\right),x\in \left[-\pi /2,\pi /2\right].$$
- By replacing *x* with x−π/2, the function $f_{2}$ is shifted to the right by π/2, thereby obtaining the function $f_{3}$ defined on the interval [0,π]:
  (7) $$f_{3}\left(x\right)=tanh\left[-s\left(x-\pi /2\right)\right],x\in \left[0,\pi \right].$$
- Since the values of $f_{3}$ at both ends of the interval [0,π] are not strictly equal to ±1, it needs to be normalized to obtain the function $f_{4}$ with a range of [−1,1]:
  (8) $$f_{4}\left(x\right)=\frac{tanh[-s(x-\pi /2)]}{tanh(s\pi /2)},x\in \left[0,\pi \right].$$
- Scale the value range of $f_{4}$ to [−0.5,0.5] and move it up by 0.5 to obtain a new annealing function Re-Tanh defined on [0,π]. The function value decreases monotonically from 1 to 0, and the shape can be adjusted by *s*. The expression is:
  (9) $$Re\text{-}Tanh\left(x\right)=\frac{tanh[-s(x-\pi /2)]}{2tanh(s\pi /2)}+0.5,x\in \left[0,\pi \right].$$

**Figure 5:**
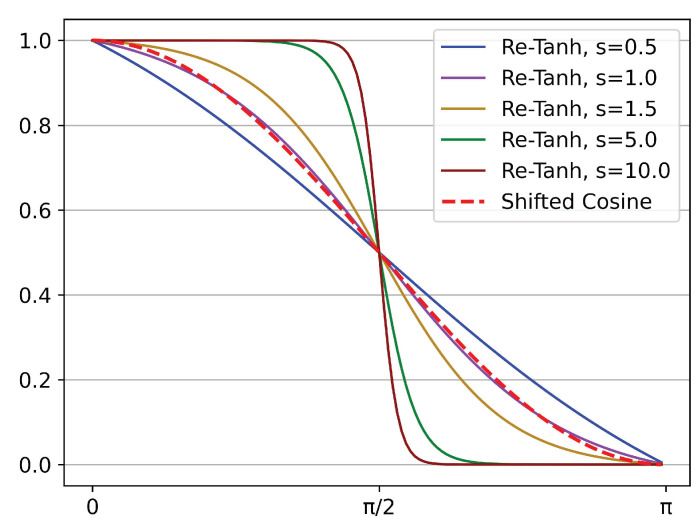
The Re-Tanh curves corresponding to different scale factors. The shifted cosine curve is also illustrated for reference.

Figure 5 illustrates the Re-Tanh curves corresponding to different *s* values. The shifted cosine is also shown for reference. It is noted that when *s* equals 1, the middle part of the Re-Tanh and the shifted cosine are almost coincident. This phenomenon can prove mathematically that the slopes of the two curves are almost equal when they are close to the center of symmetry (x=π/2), which shows that the Re-Tanh can be regarded as a generalization of shifted cosine so that the learning schedule can be flexibly adjusted in specific scenarios to improve the performance of the model. In practical applications, the *x* is usually replaced with π·mod(t,T)/T, where the mathematical quantities represented by *t* and *T* are the same as those in Equation (2).

### 3.4. Network Architecture

Compared with the task of semantic segmentation of large-scale scenes, for classification and part segmentation tasks, the processed point cloud objects are complete discrete objects, and the scale of the point cloud is smaller, so a more lightweight network can be designed. The network structure for classification and part segmentation in this paper only consists of a Local Feature Module and a Fusion Module with a global perception field. The number of points is always huge for semantic segmentation tasks, and more abstract features are required, so a deeper network consisting of a Local Feature Module and multiple cascaded Fusion Modules with enlarged local perception fields is adopted.

#### 3.4.1. Classification and Part Segmentation Network

**Figure 6:**
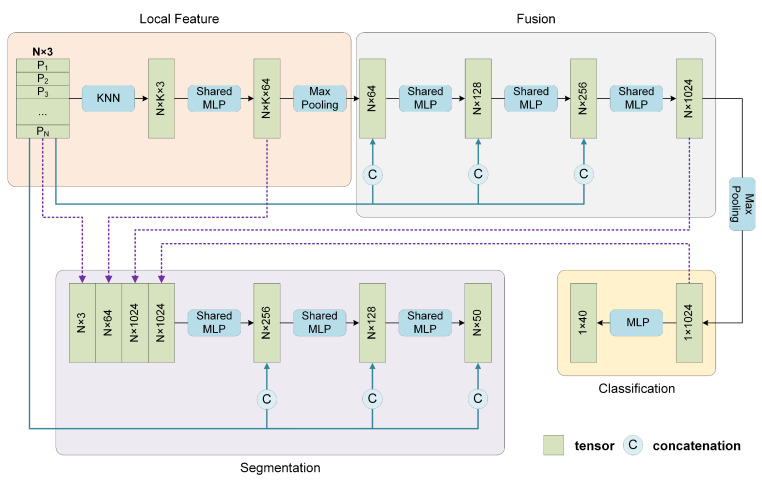
Network architecture for classification and part segmentation tasks. Some network layers are omitted for clarity.

As shown in Figure 6, the entire network mainly consists of three modules, which are used to extract the local features, fuse the local features with the supplementary global coordinates to obtain shape-level features, and perform classification or segmentation tasks, respectively. The classification network and segmentation network share the same first two modules.

For classification, first, the *N* points in the input point cloud are regarded as centroids without sampling, and then *k* neighboring points are searched. The grouped points are fed into the local feature extractor, containing three successive shared MLPs followed by a max-pooling layer to guarantee that the local features are permutation invariant. Each centroid corresponds to a group of neighboring points, and all the *N* groups share the same parameters. Subsequently, the *N* local features are sent to Fusion Module. It is noted that before each layer in the Fusion Module, the *N* local features and their *N* corresponding global coordinates are concatenated. Finally, the classification task is performed through three MLP layers. For part segmentation, the local features reflect the local geometric properties. The Fusion Module yields more abstract fusion features for each point which contain global and local information before the final max-pooling layer, and the shape-level feature acts as the output of the max-pooling layer. These features mentioned above contain complete information about an individual point: the object it belongs to, the local pattern it represents, and its spatial distribution. Then the shape-level feature is concatenated with each point’s local features and fusion features and output by the local feature extractor and Fusion Module, respectively. Then the combined feature is fed into Segmentation Module, in which the global coordinates are also provided to accomplish the segmentation tasks.

#### 3.4.2. Semantic Segmentation Network

**Figure 7:**
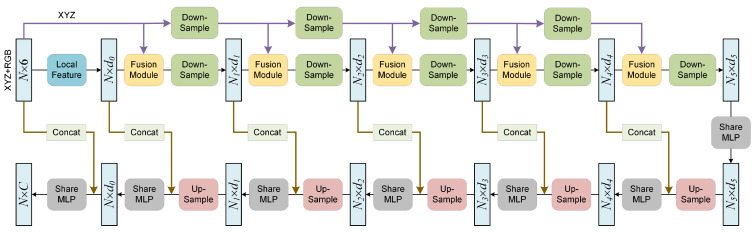
Network architecture for semantic segmentation.

In addition to the initial Local Feature Module, the semantic segmentation network mainly includes five Fusion Modules followed by down-sampling operations and five subsequent up-sampling operations. Such a design is beneficial for reducing the amount of computation and extracting more abstract features. The entire network structure is shown in Figure 7.

### 3.5. Implementation Details

The network is implemented on an NVIDIA TITAN Xp GPU with TensorFlow. For the three tasks, the batch size is set to 32, 32, and 8. For classification and part segmentation, the annealing cycle is 26 and 10 epochs, and the momentum of batch normalization (BN) starts at 0.5 and decays exponentially once every annealing cycle by a rate of 0.8. The scale factor of the Re-Tanh is set to 1.5. In each cycle, the learning rate starts at 0.01 and decreases monotonically to 0.00001. For semantic segmentation, the momentum of BN is fixed to 0.99, and a learning rate starts at 0.01 with an exponential decay rate of 0.95 per epoch is employed without ensemble technology.

## 4. Results

### 4.1. Modelnet40 Classification

Our model is evaluated on the ModelNet40 benchmark, which contains 13,834 CAD models from 40 categories divided into 9843 for training and 2468 for testing. For each object, 1024 points are uniformly sampled on the mesh surface, and then they are normalized into a unit sphere. The result is shown in Table 1. Our model achieves on-par performance with previous SOTA methods with only 1024 points as input. Note that methods such as RS-CNN [63] improve the accuracy from 92.9% to 93.6% by a tricky 10-voting strategy with randomly scaled shapes. The 10-voting evaluation is repeated 300 times, and then the best result is selected, so we take the result without voting for RS-CNN [63] for a fair comparison.

### 4.2. ShapeNet Part Segmentation

**Figure 8:**
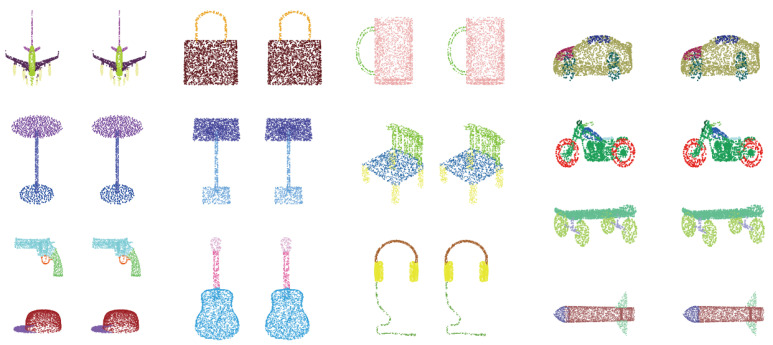
Segmentation examples of ShapeNet. For each sample, (**left column**) ground truth; (**right column**) prediction.

We evaluate the proposed model on the part segmentation task with the ShapeNet-part benchmark. The dataset contains 16,881 objects in 16 categories marked as 50 parts. For each object, 2048 points are sampled. Consistent with the previous work PointNet [27], the dataset is divided into 14,034/2847 for training/testing. The mean Intersection over Union (IoU) metric is used as a quantitative evaluation of performance, including overall instance IoU (“Instance”) and mean category IoU (“Class”). The segmentation results are presented in Table 1. Our model obtains on-par performance among the SOTA methods. Some qualitative results on the segmentation tasks are visualized in Figure 8.

### 4.3. S3dis Semantic Segmentation

Semantic segmentation of large scenes is a more complex point cloud analysis task, and Stanford 3D Indoor Space (S3DIS) dataset is utilized to evaluate the proposed model’s performance. S3DIS contains a total of 273 million points from 271 rooms in 6 large regions, and each point is represented as a 6-dimensional vector containing *XYZ* coordinates and *RGB* color, annotated with a semantic label from 13 categories. Compared with ModelNet40 and ShapeNet, which are datasets composed of discrete objects, the processing of S3DIS is more challenging in terms of the large-scale scenes.

A typical idea for preprocessing S3DIS is to divide a room into 1 m × 1 m blocks and randomly sample a fixed number of points in each block as an input to the network individually. The normalized location coordinates across the room are added to preserve the global location information for each point within the block. However, this method inevitably leads to the deterioration of spatial geometric information. A common issue is that an object is divided into two adjacent blocks, and the network outputs completely different semantic predictions for the two parts.

**Figure 9:**
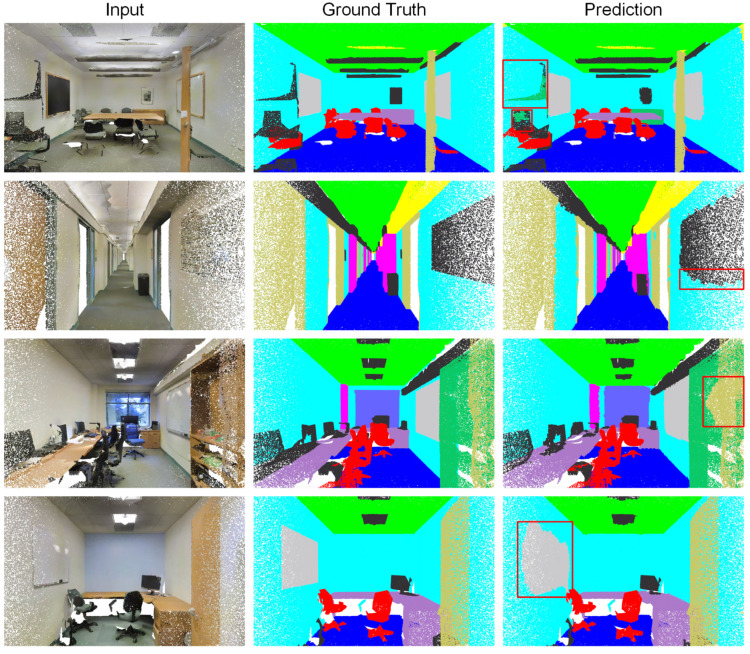
Qualitative results for the semantic segmentation task on S3DIS. Regions with large deviations between the predicted results and the ground truths are marked with red boxes.

In order to ultimately preserve the geometric information of the entire scene, we follow RandLA-Net [71] and take a large-scale scene as input. Since the local features of large-scale scenes are abstract, we extend the network used for ModelNet40 classification to a deeper one containing multiple Fusion Modules. We set RandLA-Net [71] as the baseline model. The experimental results are shown in Table 2 and Table 3, and some qualitative results are illustrated in Figure 9. Our model outperforms most of the models listed, achieving comparable performance to RandLA-Net [71]. It is worth emphasizing that, in contrast to RandLA-Net’s [71] 5-layer encoder consisting of five huge Dilated Residual Blocks, the Fusion Module of our network for S3DIS segmentation consists of only five cascaded shared MLPs, which enables our network to efficiently handle large-scale point cloud scenes while maintaining a lightweight structure. More precisely, the number of trainable parameters of our model and RandLA-Net [71] implemented on S3DIS are 1.4 million and 5.0 million, respectively.

## 5. Discussion

In this section, we first evaluate the complexity of the proposed model. Then, we perform ablation experiments on our model to prove the effectiveness of the architecture design. Finally, we conduct a series of experiments to evaluate the influence of some critical parameters. All these experiments are performed on the ModelNet40 classification task.

### 5.1. Space and Time Complexity Analysis

Table 4 summarizes the space (number of parameters in the proposed model) and time (floating-point operations per sample) complexity. While PCNN [48] and PointNet [27] exhibit higher performance in computational cost (measured in FLOPs/sample), the proposed model is still quite efficient. The proposed model contains the least number of parameters among the listed point-based models. Compared with PointNet [27] and PointNet++ [28] which are also based on shared MLP, the number of parameters is reduced by a large margin of 72.0% and 34.7%.

### 5.2. Ablation Study

The feature extractor used in our model is shared MLP, which seems a little simple compared to some complex and sophisticated feature extractors. However, our model shows good performance, primarily due to the supplement of spatial distribution information and the proposed learning schedule. We study the validity of these components, and the results are shown in Table 5. The “Part. supple.” means that the spatial coordinates are supplemented to the features only once in the first layer of the Fusion Module. The “Supple.” means the coordinates are supplemented with each layer in the Fusion Module. Furthermore, the “Cosine” and “Re-Tanh” imply that the model adopts the snapshot ensemble with cosine and the proposed Re-Tanh annealing schedules. For models such as Model A, B, and C without ensemble strategy, the learning schedules adopt an exponential decay learning rate that starts at 0.001 and decreases by 0.5 every 26 epochs, consistent with the annealing cycle.

The baseline Model A only obtains an accuracy of 90.9%, and the accuracy of Model B is significantly improved to 92.6% with the supplementary information, but still lower than the 93.5% obtained by our model. Model A only considers local features without their locations, and the shape-level feature finally obtained is incomplete. Model B attempts to contain the spatial information of local features in the inference, but coordinates are fed into only one layer. Although the performance is improved significantly compared with Model A, Model B trivially supplements the distribution information to the local features only once. Model C takes the spatial coordinates as supplements for local patterns in each layer in the Fusion Module and achieves an accuracy of 92.9%. Comparing Model A and C, it can be verified that the supplement of spatial information is critical to improving the performance significantly. Model D adopts a cosine annealing strategy for snapshot and achieves 93.0% accuracy, and the improvement is not significant compared with Model C (92.9%). The model (Ours) with the proposed Re-Tanh annealing strategy outperforms all these models above, especially Model D, proving that an adjusted annealing learning schedule is essential for better performance.

### 5.3. Scale Factor

The proposed learning schedule can adjust the learning schedule flexibly to make the model adaptive to various scenarios. To evaluate the effectiveness of the Re-Tanh, we conduct a series of experiments corresponding to different scale factors. The results are shown in Table 6, and the result corresponding to cosine annealing is also illustrated for reference. For ModelNet40 classification, the model achieves the highest accuracy when *s* is set to 1.5, proving that the Re-Tanh is more flexible and more applicable than cosine annealing.

### 5.4. Point Density

**Figure 10:**
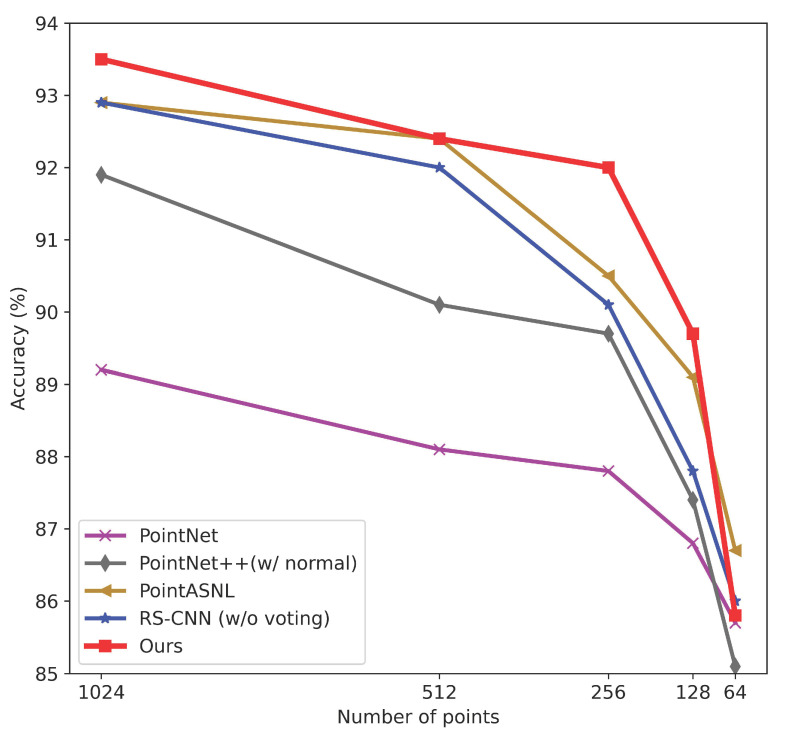
Accuracy at different densities.

Although 1024 points per shape can be sufficient to extract discriminative features, the number of points sampled from a shape cannot always be enough and uniform in real scenarios. To evaluate the performance on different densities, we randomly sample points from the 2048 points of each shape for training and evaluation. In order to keep the neighborhood area constant, the number of neighbor points changes in the same proportion as the density changes. Precisely, 1024 points correspond to the number of neighboring points *k* equals 48. Thus, when the numbers of sampling points are 512, 256, 128, and 64, *k* is set to 24, 12, 6, and 3, accordingly. The result is shown in Figure 10. Even in the case of 128 points, our model can still achieve an accuracy of 89.7%, which exceeds 89.2% of PointNet [27] with 1024 points as input. This result shows that the proposed method can extract discriminative features in extremely sparse points, making the network highly adapted to various point densities.

### 5.5. Neighborhood Size

A proper neighborhood size is crucial for the local features. The number of neighboring points is denoted as *k*. Moreover, experiments are performed to evaluate the robustness to the variation of it. The results are shown in Table 7. It can be seen that the highest accuracy is achieved when k=48. The reason is that when *k* is too small, the proposed model cannot extract generally representative local patterns. On the contrary, when *k* is too large, the extracted local features contain too much information that is not closely related to the local regions. Although the changes in neighborhood size can affect the performance of the proposed model, it is worth noting that the model still maintains high accuracy.

### 5.6. Reduced Training Dataset

In many scenarios, the labeled samples are expensive and not always sufficient. To evaluate the feature extraction capability of the proposed model, we set a series of reduced training data. Precisely, in the ModelNet40 data provided by [27], the training set consists of five files in total, the first four files each contain 2048 shapes, and the last file contains 1648 shapes. The distribution of ModelNet40 training data in each category is unbalanced, and in each file, this imbalance will be more serious. We directly adopt each file as a reduced training set, and the results are illustrated in Table 8. It can be seen that the proposed model outperforms PointNet [27] on each reduced training set by a large margin, which implies that the proposed model can maintain an effective feature extraction capability on a small unbalanced training dataset.

## 6. Conclusions

This paper studies the limitation of MLP-based methods and proposes a lightweight architecture for point cloud analysis. Inspired by CNN, we propose the supplementary mechanism of distribution information for shared MLPs and perform it concisely. Moreover, the model converges rapidly with an exponential decay learning rate, so we promote it with the snapshot ensemble strategy and design a new cyclic annealing schedule that can be flexibly adjusted. Our network achieves on-par performance with the previous SOTA methods, with the least number of parameters. Although effective, there are also some limitations. The kNN search is performed for each point when performing classification tasks resulting in overlapping points between the local regions of adjacent centroids, which makes the computation redundant. Adaptive learning of a subset of keypoints in a point cloud and designing more effective architectures for efficient point analysis will be explored in our future studies.

## Figures and Tables

**Table 1:** Shape classification and part segmentation results (%) (“nor”: normal; “-”: unknown; “k”: 1024). The best scores in the table are marked in bold font.

	ModelNet40		ShapeNet		
Method	Input	Acc.	Input	Cls. mIoU	Ins. IoU
PointNet [27]	1 k	89.2	2 k	80.4	83.7
SCN [64]	1 k	90.0	1 k	81.8	84.6
KD-Net (depth = 10) [19]	1 k	90.6	4 k	77.4	82.3
PointNet++ [28]	1 k	90.7	2 k, nor	81.9	85.1
KCNet [65]	1 k	91.0	2 k	82.2	84.7
MRTNet [66]	1 k	91.2	-	-	-
SpecGCN [67]	1 k	91.5	2 k	-	85.4
KD-Net (depth = 15) [19]	32 k	91.8	-	-	-
PointCNN [68]	1 k	92.2	-	-	-
PCNN [48]	1 k	92.3	2 k	81.8	85.1
DGANet [51]	1 k	92.3	2 k	-	85.2
Point2Sequence [69]	1 k	92.6	-	-	-
A-CNN [49]	1 k	92.6	-	-	-
Hybrid-CNN [59]	1 k	92.6	-	-	-
DGCNN [30]	1 k	92.9	2 k	82.3	85.1
RS-CNN w/o vot. [63]	1 k	92.9	2 k	**84.0**	86.2
Point2Node [56]	1 k	93.0	-	-	-
GAPointNet [53]	1 k	93.0	2 k	-	84.9
PCT [33]	1 k	93.2	2 k	-	**86.4**
Point2SpatialCapsule [55]	1 k	93.4	2 k	83.0	85.3
AGNet [70]	1 k	93.4	2 k	82.7	85.4
Ours	1 k	**93.5**	2 k	82.7	85.7

**Table 2:** Results of 6-fold cross validation on the S3DIS dataset (%). The best scores in the table are marked in bold font.

Method	mIoU	OA	mAcc
PointNet [27]	47.6	78.6	66.2
SL-Ontology [72]	49.9	-	-
PointNet++ [28]	54.5	81.0	67.1
DGCNN [30]	56.1	84.1	-
RSNet [73]	56.5	-	66.5
AGNet [70]	59.6	85.9	-
PointCNN [68]	65.4	**88.8**	75.6
PointWeb [47]	66.7	87.3	76.2
RandLA-Net [71]	**70.0**	88.0	**82.0**
Ours	68.7	87.5	81.2

**Table 3:** Results of S3DIS Area-5 (%). The best scores in the table are marked in bold font.

Method	mIoU	mAcc
PointNet [27]	41.09	48.98
PointCNN [68]	57.26	63.86
PointNet++ [28]	57.27	63.54
PointWeb [47]	60.28	66.64
RandLA-Net [71]	60.63	68.81
MinkowskiNet [39]	**65.35**	**71.71**
Ours	61.38	70.68

**Table 4:** Complexity of the model for ModelNet40 classification (res: resolution; k: 1024; M: million). The three parts from top to bottom are voxel-based, view-based and point-based methods.

Method	Params	FLOPs/Sample	Input	Acc. (%)
VoxNet [16]	0.8 M	-	30×30×30 voxel	83.0
MVCNN [22]	60.0 M	62,057 M	80 views	90.1
KCNet [65]	0.9 M	-	1k points	91.0
PointNet++(SSG) [28]	1.5 M	1684 M	1 k points	90.7
PointNet++(MSG) [28]	1.7 M	4090 M	1 k points	91.9
DGANet [51]	1.7 M	-	1 k points	92.3
DGCNN [30]	1.8 M	2430 M	1 k points	92.9
GAPointNet [53]	1.9 M	1228 M	1 k points	93.0
AGNet [70]	2.0 M	-	1 k points	93.4
KD-Net (depth = 15) [19]	2.0 M	-	32 k points	91.8
SpecGCN [67]	2.0 M	1112 M	1 k points	91.5
PCT [33]	2.9 M	2320 M	1 k points	93.2
PointNet [27]	3.5 M	440 M	1 k points	89.2
PCNN [48]	8.2 M	294 M	1 k points	92.3
Ours	0.98 M	968 M	1 k points	93.5

**Table 5:** Ablation studies of the proposed model (%).

Model	Local	Part. Supple.	Supple.	Cosine	Re-Tanh	Acc.
A	✓					90.9
B	✓	✓				92.6
C	✓		✓			92.9
D	✓		✓	✓		93.0
Ours	✓		✓		✓	93.5

**Table 6:** Results corresponding to different scale factors (%).

s	0.5	1.0	1.5	2.0	2.5	Cosine
Acc.	93.0	93.1	93.5	93.1	92.9	93.0

**Table 7:** Results of different sizes of local regions (%).

Size	12	24	36	48	60
Acc.	92.3	92.4	92.7	93.5	93.1

**Table 8:** Results on reduced training set (%).

Method	File-0	File-1	File-2	File-3	File-4	All Files
PointNet [27]	79.2	81.8	81.2	79.3	78.8	89.2
Ours	88.3	88.7	88.5	88.8	87.3	93.5

## Data Availability

ModelNet40: https://shapenet.cs.stanford.edu/media/modelnet40_ply_hdf5_2048.zip; ShapeNet part: https://cs.stanford.edu/~ericyi/project_page/part_annotation/index.html; S3DIS: https://goo.gl/forms/4SoGp4KtH1jfRqEj2, accessed on 8 October 2021.

## Abbreviations

The following abbreviations are used in this manuscript:

CNN	Convolutional neural network
MLP	Multi-layer perceptron
SOTA	State-of-the-art
3D	Three-dimensional
2D	Two-dimensional
Re-Tanh	Rectified-Tanh
kNN	K nearest neighbor
GPU	Graphics processing unit
BN	Batch normalization
S3DIS	Stanford 3D Indoor Space
FLOPs	Floating-point operations
